# Mania for Novelties in Medicine

**Published:** 1887-06

**Authors:** 


### Mania for Novelties in Medicine,

Probably there is to be found no period in the past history of medicine, characterized by such an active strife for making new discoveries, not only in the prolific realm of bacteriology, but in new therapeutic inventions and in novel applications of old remedies, as is the present. The mania for discoveries and inventions and the thirst for novelties have became fairly epidemic. It is only necessary to invent some kind of apparatus and either some new remedy or some new combination of old ones, apply them to the treatment of a few cases of some dangerous form of disease, or, still better, to disease in such stage of advancement as has been regarded hopeless, and report some striking improvement, before time enough has elapsed to make permanent results possible, and it is at once heralded as a grand achievement. It is so important that it cannot wait for the slow circulation of the medical press, but quickly reaches the profession and the public simultaneously through the daily secular press and telegraph. And now comes the strife between the ambitious doctors to be first in the field with the new remedies, and if the more considerate would like to delay until more reliable results could be possible their patients speedily compel them to fall into line, or abandon them for those who have succeeded in first becoming armed and equipped for the new method of miraculous healing, until the manufacturers are hardly able to supply the needed materials.

It makes no difference whatever, whether the morbid conditions to be cured are lungs containing one hundred suppurating cavities varying from the size of a pea to that of a hen's egg, and the mucous membrane of the bowels ragged with ulcerations, if a temporary improvement can be maintained for two or three weeks, is sufficient to constitute the subject of another favorable report, and give an additional impetus to the strife. And yet, as inexorable time moves on just as many thousands of dollars have become invested in the new material, and the hopes of many thousands of invalids have been revived, the stubborn fact remains, that cancers will not soften and disappear under the influence of condurango, nor a continued fever flee *for good* from a cold bath or a dose of antipyrin, nor a half disorganized lung be reproduced, good as new, in three or four weeks by the magic influence of gaseous enemas. The many medical friends who have written for our *candid* opinion of the value of the Bergeon treatment of consumption, as well as those who are intending to write may gather from the foregoing our answer; which is, that it is a method of treatment as capable of doing harm, unless skillfully used, as it is of doing good ; and that from the known pathological and anatomical changes involved in all the varieties of pulmonary phthisis, it is not possible to determine the real value of this or any other treatment until a much longer time has elapsed. —*Journal American Medical Association.*

